# News and Notes

**Published:** 1903-04

**Authors:** 


### NEWS AND NOTES.

(We are indebted to the *Medical Age* for many of these notes.)

---

THE health officials of Mobile have discovered eleven cases of smallpox among the students of the Medical College of Alabama, Mobile.

---

A BILL has been introduced in the house appropriating $20,000 for the renovation of the building of the Medical College of Alabama, Mobile.

---

THE suggestion is made that the name of the late Dr. Alexander J. C. Skene be perpetuated by the naming of some suitable avenue in Brooklyn.

---

ON March 4 the senate of Indiana defeated Senator Johnston's bill, providing a separate board of examination and registration for osteopaths of the State.

---

IN a trial at Atlanta, March 3, the fact was elicited that at one drug store in that city more than 3,000 prescriptions for cocain had been filled within two months.

---

THE new operating-room at the City Hospital, Huntsville, erected and equipped by the City Council in memory of the late Dr. R. Barnwell Rhett, Jr., was recently opened.

---

DR. EDWARD SOUCHON, New Orleans, has been reappointed President of the State Board of Health, and Dr. Joseph S. Stephens, Natchitoches, and Percival B. McCutcheon, New Orleans members.

CONGRESS has passed a bill appropriating $125 per month during her lifetime as a pension to Mrs. Emily Lawrence Reed, widow of the late Major Walter Reed, U. S. A.

---

BY the will of the late A. C. Hutchinson, the bulk of his estate is devised to Tulane University Medical Department. The estate is appraised at $991,169. The brother of the deceased has begun suit at New Orleans to break the will on the ground that the university is not competent to receive the bequest.

---

DR. H. R. GAYLARD recently made a gastrostomy on a patient at the Erie County Hospital, when the following articles, numbering 689 and weighing 2 pounds and 3 ounces, were removed from the stomach; One piece of chain 3 inches in length, 41 knife blades, 2 wire nails, 142 screws, 3 nuts, 452 sharp tacks, 48 shoemaker nails, 144 grammes of glass.

---

NINE of the trained nurses of the New York Eye and Ear Infirmary left the hospital on the refusal of Dr. Richard H. Derby, the executive surgeon, to accede to their demand that the new head nurse and an objectionable assistant be removed. For a short time the service of the hospital was badly crippled by this high-handed action, but several nurses were sent from other institutions to render assistance in the emergency.

---

IN the suit brought against Drs. John A. Wyeth and W. H. Landon White by Mrs. Alice Wood, as administratrix of the estate of her son, Robert Wood, deceased, for damages sustained by him by reason of alleged negligent treatment, the Appellate Division of the Supreme Court dismissed the case on its merits. The boy, who was being operated on by Dr. Wyeth for an infected wound of the arm, died under chloroform, and suit was thereon instituted. It will probably be carried to the appellate court.

---

APPLICATIONS are pouring in on the Governor for appointment to scholarships in Augusta Medical College, under the law whereby the Governor is authorized to name two persons from each of the eleven congressional districts of the State for free scholarships at this institution.

---

THE annual report of the Charity Hospital, New Orleans, shows revenues for the year of $13499,7.14, and expenditures of $126,-042.15, leaving a balance of $8,756.99. During the year the hospital received $34,895.21 from donations and legacies. There were 6,793 patients admitted during the year.

---

THE Supreme Court has confirmed the decision of the lower court for the defendants in the case of E. S. E. Bryan *versus* The Atlanta Society of Medicine. Bryan brought action against the society for $10,000 damages on the ground that his name had been presented to the grand jury as an illegal practitioner of medicine.

---

DR. O. H. SNIDER, Atlanta, arraigned on the charge of distributing free packages of cocain to negroes, was found guilty, February 25, and fined $500, a thirty days' sentence in the stockade, and bound over to the City Court on a $1,000 bond. On his promise not again to offend the fine was reduced to $100. W. C. Moran, Atlanta, on March 3, was fined $100 and costs for dispensing cocain in a box which did not bear the red label required by law.

---

DR. T. CHILTON THORINGTON, Montgomery, has been appointed captain and assistant surgeon, S. T. Ala., and assigned to the Second Infantry, vice Dr. Walter S. Britt, Eufaula, resigned.

Dr. Henry M. Hunter, Union Springs, has located in the City of Mexico. Dr. William M. Faulk, who has been a member of the staff of Bryce Hospital for the Insane, has resigned, and will practice in Tuskaloosa.

---

A MEETING was recently called in Philadelphia by the International League of Barbers to further the passage of a bill by the Pennsylvania Legislature providing sanitary regulation of barber shops. The proposed law provides for the licensing and registration of barbers, and requires their better education. It also provides for a board of examiners, of five members, who shall have been actually engaged in the business at least ten years. The movement is supported by the International Union of Journeymen Barbers.

---

DR. FLEXNER is engaged in manufacturing his dysentery antitoxin, or "bacteriolysin," as he terms it. Trial will be made of this antitoxin this summer at the Wilson Sanitarium near Baltimore. In about two months Dr. Flexner will institute a course of instruction for about thirty young men at his laboratory in Philadelphia, on the identification of the dysentery bacillus, so that during the summer a very widespread study of the summer diarrheas of children in this country may be made. It is rumored that Dr. W. G. McCollum, associate professor of pathology at the Johns Hopkins Medical School, may succeed Dr. Flexner at the University of Pennsylvania.

